# Hydroponic Forage in Ruminant Nutrition: A Systematic Review of Nutritional Value, Performance Outcomes, and Sustainability

**DOI:** 10.3390/ani15243544

**Published:** 2025-12-09

**Authors:** Alessandro Vastolo, Monica Isabella Cutrignelli

**Affiliations:** Department of Veterinary Medicine and Animal Production, University of Naples Federico II, 80137 Naples, Italy; alessandro.vastolo@unina.it

**Keywords:** hydroponic forage, sprouted barley, wheat sprouts, ruminant nutrition, digestibility, milk yield, methane emissions

## Abstract

This systematic review evaluated 28 peer-reviewed studies on the use of hydroponic forage in ruminant diets, following PRISMA 2020 guidelines. Hydroponic barley sprouts contain on average 14.8 ± 2.1% CP, 3.6 ± 0.4% EE, 12.9 ± 1.7% NDF, 7.8 ± 1.2% ADF, and 10.5 ± 2.8% DM (mean ± SD; n = 21 studies), and mineral content, though limited by high moisture and low dry matter yield. Among the included studies, 61% focused on barley, confirming its suitability for hydroponic cultivation. In dairy cattle, hydroponic forage improved milk fat content and oleic acid (C18:1), linoleic acid (C18:2), and α-linolenic acid (C18:3) and reduced saturated fatty acids without compromising yield. In buffaloes, inclusion enhanced cheese quality and reduced energy footprint, though costs were higher. For small ruminants and growing animals, moderate inclusion (5–25% dry matter) improved intake, digestibility, and growth, while excessive replacement reduced feed intake or digestibility, likely due to rumen microbiota shifts. Additionally, hydroponic feeding reduced methane emissions in lambs, highlighting its environmental potential. Overall, hydroponic forage can serve as a sustainable complement to conventional feed resources, promoting resource efficiency and animal performance when properly integrated into balanced diets. Further studies should define optimal inclusion rates and evaluate economic and environmental trade-offs under different production systems.

## Simple Summary

Hydroponic forage is a type of feed grown without soil, using only water and controlled environmental conditions. It is produced mainly from cereals such as barley, maize, and wheat, which sprout within a few days and create a fresh, nutritious plant material that can be fed to cattle, buffaloes, sheep, and goats. This review examined 28 scientific studies to understand how hydroponic forage affects animal nutrition, productivity, and sustainability. The results show that hydroponic forage can improve milk quality, growth rate, and feed efficiency. It also requires much less land and water than traditional forage crops, making it interesting for areas affected by drought or limited soil resources. However, hydroponic forage has some weaknesses, such as high production costs, high moisture content, and lower dry matter yield, meaning that animals must eat larger amounts to obtain the same nutrients. Overall, hydroponic forage is not a full replacement for conventional feed, but it can be a useful complementary ingredient when included at moderate levels. More research is needed to define the most cost-effective inclusion rates and to better evaluate long-term environmental and economic effects.

## 1. Introduction

The term “hydroponics” refers to a soilless cultivation technique that enables plant growth using aqueous solutions enriched with essential nutrients [1]. Historically applied in horticulture and floriculture, hydroponics has recently gained prominence in the field of animal nutrition, particularly in the production of forage for ruminants [2]. This application, known as hydroponic green forage (HGF), involves the germination of cereal or legume seeds under controlled environmental conditions, without the use of soil, producing a nutrient-rich and highly digestible biomass [2,3]. The motivation behind this innovation lies in the limitations of conventional forage production, which depends on large land areas and is vulnerable to pedoclimatic variability. Hydroponic forage systems have been proposed as a complementary solution, particularly in regions with limited access to arable land and water. These systems, ranging in scale and automation, rely on rapid indoor germination of cereal seeds, mainly barley, and the entire growth cycle typically spans 7 to 10 days [4]. Fresh biomass yield is substantial, and water usage is reduced by up to 90% compared to traditional cultivation methods [3]. Its chemical composition is particularly suitable for ruminant nutrition: dry matter content ranges from 10 to 15% on a fresh basis, crude protein from 14 to 20% of dry matter (DM), and ether extract from 3 to 4%. Structural carbohydrates are lower and more digestible than in conventional forages (neutral detergent fiber, NDF: 12–13%; acid detergent fiber, ADF: 6–11% of DM), and mineral concentrations increase during germination (Ca: 0.2–0.3%; P: 0.3–0.4%). Meanwhile, metabolizable energy averages 12 MJ/kg DM (2.8 Mcal/kg) [5]. Furthermore, HGF is rich in micronutrients and enzymatic compounds (e.g., vitamin E, α-amylase) that may enhance digestibility and nutrient absorption. Hydroponic forage has been successfully tested in cattle, buffalo, sheep, and goats, showing positive effects on palatability, digestibility, and, in some cases, milk production and meat quality [5]. The main advantages of hydroponic forage systems include high water-use efficiency, reduced need for pesticides and fertilizers, year-round production independent of climatic conditions, and minimal land requirement [2,3]. Environmentally, hydroponics produces less waste, mitigates soil erosion, and decreases greenhouse gas emissions compared to traditional agriculture [5]. Nevertheless, hydroponic systems face several limitations. Chief among them are high initial capital costs, particularly for climate-controlled and automated units, and the need for technical expertise in nutrient solution preparation and environmental monitoring [3]. Additionally, HGF has a very short shelf life and must be fed fresh to preserve its nutritional value. While nutritionally rich, hydroponic forage may lack structural fiber, necessitating dietary adjustments to maintain optimal rumen function. Some studies also report conflicting results regarding productive performance, indicating the need for further research and standardization in cultivation and feeding protocols [6,7].

A systematic review is defined as a rigorous and transparent method of evidence synthesis in which relevant studies are systematically identified, screened, and analyzed according to predefined inclusion criteria and standardized procedures. This approach reduces selection bias, enhances reproducibility, and allows a comprehensive and critical evaluation of the effects of hydroponic forage across ruminant species. Such an approach is particularly relevant for hydroponic forage, as the available studies differ widely in crop species, cultivation systems, inclusion levels, and measured outcomes. A systematic review enables these heterogeneous findings to be compared and interpreted consistently, providing a clearer understanding of the true nutritional value, productive effects, and limitations of hydroponic forage in ruminant diets.

## 2. Materials and Methods

This systematic review was conducted following the PRISMA 2020 guidelines. PRISMA (Preferred Reporting Items for Systematic Reviews and Meta-Analyses) is an evidence-based set of standards designed to improve the transparency, reproducibility, and methodological rigor of systematic reviews. It provides structured recommendations for literature search, study selection, data extraction, and reporting, ensuring that the synthesis of evidence is comprehensive and unbiased [8].

**Search Strategy**

The systematic review was conducted according to PRISMA 2020 guidelines. A comprehensive search was performed in Scopus, Web of Science, PubMed, and Google Scholar between 30 May and 30 June 2025, using the terms “hydroponic forage”, “sprouted barley”, “hydroponic wheat”, “hydroponic maize”, “fodder”, and “ruminants”.

**Eligibility Criteria**

Studies were included when they (i) involved ruminants, (ii) evaluated hydroponic forage (barley, wheat, maize, or oat), (iii) reported at least one outcome related to growth, milk production, digestibility, rumen fermentation, blood parameters, or methane emissions, and (iv) used a controlled in vivo design. Reviews, in vitro studies, conference abstracts, and incomplete reports were excluded.

**Study Selection**

**Figure 1:**
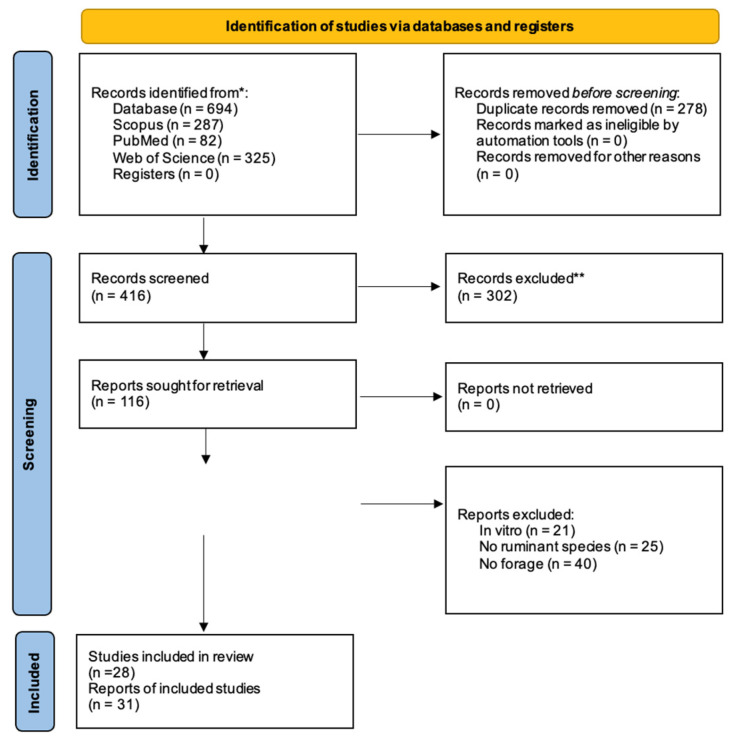
PRISMA 2020 flow chart for new systematic reviews that include only searches of databases and registries. * Consider, if feasible to do so, reporting the number of records identified from each database or register searched (rather than the total number across all databases/registers). ** If automation tools were used, indicate how many records were excluded by a human and how many were excluded by automation tools.

Two independent reviewers screened titles, abstracts, and full texts. Disagreements were resolved by consensus. The PRISMA flowchart summarizes the process (Figure 1).

**Data Extraction**

From each study we extracted species, breed, type of hydroponic crop, inclusion level (% DM), diet composition, dry matter intake (DMI), growth performance, milk yield and composition, digestibility coefficients, rumen fermentation parameters (pH, ammonia, NH_3_-N, volatile fatty acids, and VFA), blood metabolites, and methane emissions.

## 3. Overview of the Final Article Selected

**Figure 2:**
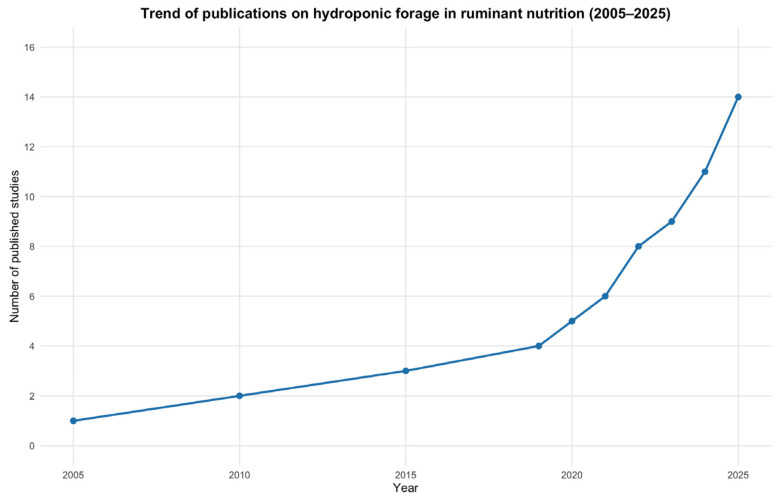
Temporal trend of scientific publications on hydroponic forage in ruminant nutrition (2005–2025).

A total of 28 peer-reviewed articles met the predefined inclusion and exclusion criteria and were selected for this systematic review. Table 1 summarizes the main characteristics of the included studies. The earliest research on the use of hydroponic forage in animal nutrition dates back to 2009, with a single study published in that year. In 2016, there was a gradual increasing trend in the number of publications, followed by a more pronounced rise after 2018 (Figure 2). Notably, 2024 marked a substantial surge in research activity, with 10 articles published in that year alone, indicating a growing scientific interest in hydroponic forage applications for ruminant feeding. This positive trend appears to be ongoing into 2025, with two studies already published in the first half of the year. This temporal distribution suggests a renewed and expanding focus on alternative forage systems, likely driven by increasing concerns over climate resilience, feed security, and sustainability in animal production systems.

In terms of geographic distribution of the studies included in this systematic review, China emerged as the leading contributor, with six studies, followed by Italy and India, each with five publications. Indonesia and the United States contributed two studies each, while several other countries, including Malta, Pakistan, Egypt, Mexico, South Korea, Ethiopia, Iran, and South Africa, each contributed one study (Table 1).

This distribution reflects a wide international interest in hydroponic forage research, spanning both developing nations, where such systems may help address limitations related to land availability, water scarcity, or climatic challenges, and industrialized countries like Italy and the United States, where hydroponics is increasingly integrated into sustainable and innovative livestock production strategies.

Among the 28 studies included in this systematic review, the most frequently used hydroponic crop was barley (*Hordeum vulgare*), featured in 17 studies (60.7%), confirming its role as the most extensively studied species in ruminant nutrition. Maize (*Zea mays*) was the second most used crop, appearing in 6 studies (21.4%), while wheat (*Triticum* spp.) was used in 4 studies (14.3%). Oat (*Avena sativa*) was reported in only two studies (7.14%). These results highlight a clear predominance of barley as the primary choice for hydroponic forage production in ruminants, likely due to its high germination capacity, rapid growth, and favorable nutritional composition.

The majority of the studies (43%) included in this systematic review focused on growing animals, specifically calves and lambs. Calves were investigated in five studies (17.9%), while lambs were the subject of six studies (21.4%), highlighting the importance of these young animals in intensive farming and nutritional trials. Sheep were represented in three studies (10.7%) and goats in four studies (14.3%), reflecting increasing scientific interest in the use of hydroponic forage in small ruminant nutrition. Adult cows were the subject of seven studies (25%), and adult buffaloes in three studies (10.7%), with buffalo calves included in one study (3.6%). Overall, this distribution indicates a predominant focus on cattle and young animals, underlining production priorities and the interest in evaluating alternative feeding strategies during critical growth phases.

## 4. Nutritional Characteristics of Hydroponic Fodder

Of the 28 studies included in this review, 21 (75%) reported chemical composition data for hydroponic cereal forages. The descriptive statistics of the main nutritional parameters (mean, SD, minimum, and maximum values) for barley, maize, and wheat are presented in Table 2. Overall, the chemical composition of hydroponic forages shows substantial variability, reflecting differences in crop species, seed quality, growth duration, and environmental conditions across studies.

Barley is the most frequently investigated crop and shows a relatively balanced composition, with moderate protein and ether extract contents, intermediate levels of structural carbohydrates, and appreciable amounts of non-structural carbohydrates such as starch. Maize exhibits similar dry matter levels but is characterized by slightly lower crude protein and higher ether extract content, together with greater NSC concentrations, suggesting a higher potential energy yield. Wheat, although represented by fewer studies, generally presents higher dry matter and crude protein contents and the lowest fiber fractions among the three cereals.

One additional study reported the chemical composition of hydroponic oats, although only limited nutrient information was available, and, therefore, these data were not included in the comparative table.

**Figure 3:**
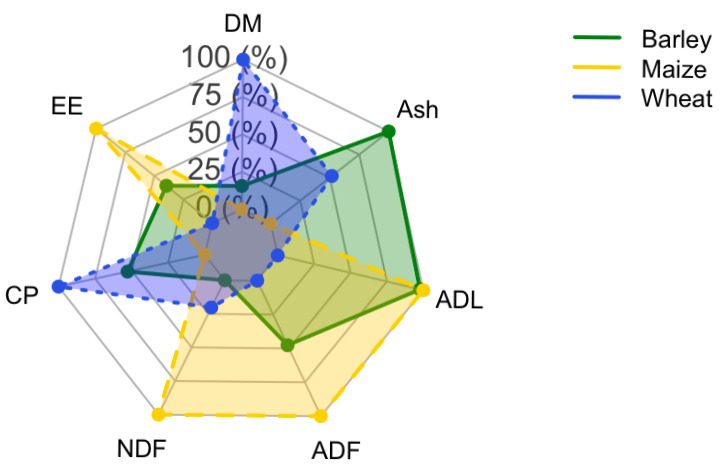
Comparative nutritional profile of three hydroponic crops (barley, maize, and wheat) expressed as a percentage on a dry matter basis. The axes represent key chemical components: dry matter (DM), ether extract (EE), crude protein (CP), neutral detergent fiber (NDF), acid detergent fiber (ADF), lignin (ADL), and ash. Colored lines indicate the crops, while the maximum and minimum values for each parameter are shown as reference. The radial scale values (0, 25, 50, 75, 100%) indicate the relative percentage of each component, normalized to the maximum value observed among the analyzed crops.

Figure 3 illustrates the relative chemical profiles of barley, maize, and wheat using a radar chart. The graphical representation highlights the distinct nutritional patterns of the three cereals: barley with a more balanced distribution, maize with higher lipid and fiber fractions, and wheat with greater dry matter and protein and lower fiber content. Despite these inter-crop differences, all hydroponic forages share a common limitation: their low dry matter content, which reduces nutrient density on an as-fed basis.

## 5. Dairy and Adult Ruminants

The use of hydroponic forage in adult ruminants has been investigated mainly for its effects on milk production and quality. Early evidence from Agius et al. [6] showed that replacing conventional forage with hydroponically grown barley increased milk fat concentration by approximately 3–5%, while milk pH rose by about 1%. Protein and lactose levels remained unchanged. These results suggest a modest but consistent improvement in selected milk quality traits, supporting further investigation into the role of hydroponic forage in dairy production systems. Zang et al. [23] evaluated the partial replacement (10%) of conventional concentrates with hydroponic barley or wheat sprouts. Their results showed that sprouted barley improved feed efficiency, both in terms of milk yield/DMI and ECM yield/DMI, whereas sprouted wheat enhanced body weight gain. Moreover, the apparent total-tract digestibility of nutrients was higher for sprouted wheat than for sprouted barley. Overall, the inclusion of hydroponic sprouts did not impair milk yield and demonstrated specific nutritional advantages depending on the cereal species used.

Hydroponic forage has been widely evaluated in dairy ruminants, particularly for its effects on milk yield, milk composition, and feed efficiency. Across studies, moderate inclusion rates generally showed neutral or slightly positive effects, whereas high inclusion often reduced dry matter intake (DMI) due to the high moisture content of sprouts.

Fazaeli et al. [5] reported that including hydroponic barley at 10–20% DM of the diet (equivalent to replacing 20–60% of the corn silage on a fresh basis) did not affect milk yield or milk fat, protein, or solids, with variations remaining within ±2% of control values. Feed efficiency also remained unchanged. These results indicate that hydroponic barley can replace a portion of the forage without compromising milk performance, although its relatively low DM yield implies higher production costs.

Wu et al. [22] demonstrated that the effect of hydroponic barley depends on dietary protein balance. When included at 4.8% DM within a high-protein diet (16.8% CP), hydroponic barley increased milk yield by ~4% and milk protein yield by ~3–5%, while under a low-protein diet (15.5% CP) it reduced DMI by 5–8% without improving milk output. These findings highlight that the response to hydroponic forage is modulated by the nutritional context of the diet.

Ceci et al. [16] evaluated hydroponic barley produced using treated urban wastewater and found that a 5% DM inclusion neither impaired health nor altered milk production. Milk yield and composition varied by less than ±1% compared with the control diet, confirming that low-level inclusion is safe and potentially beneficial for water-saving systems in arid regions. Evidence from studies on heifers confirms that hydroponic sprouts can be used as a partial substitute for conventional concentrate ingredients. Kim et al. [25] showed that replacing 10–30% of cornmeal with hydroponically sprouted barley did not affect growth rate or blood metabolites, with body weight gain differing by less than ±3% between treated and control groups. Ren et al. [21] found that high inclusion of whole-plant hydroponic barley (40–50% DM) reduced DM digestibility by 6–8% and CP digestibility by 4–6%, while shifting the rumen microbiota toward higher *Lachnospiraceae* and lower *Ruminococcus* abundance. In contrast, moderate inclusion (10–25% DM) improved total VFA production by 12–18%, consistent with enhanced fermentative efficiency.

Research on buffaloes, all occurring within the FORIDRO project, provides species-specific insights. Balivo et al. [10,24] demonstrated that hydroponic barley can be detected in raw milk using an electronic nose system and that mozzarella from hydroponic-fed buffaloes displayed lower hardness (−5 to −10%) and a more favorable fatty acid profile, indicating improved cheese quality. Masucci et al. [4] reported that replacing silage with hydroponic barley increased milk yield slightly (+3–4%) and reduced the water footprint but substantially increased production costs due to the low DM yield of hydroponic barley. This suggests that hydroponic forage may offer qualitative and environmental advantages but requires careful economic evaluation in commercial buffalo production (Table 3).

## 6. Small Ruminants

Hydroponic forage has also been evaluated extensively in small ruminants, especially goats, where several studies report improvements in growth, reproductive traits, and feed efficiency. In Black Bengal goats, Rajak et al. [7] showed that combining hydroponically grown maize and wheat increased average daily gain by 10–15%, improved reproductive performance, and reduced feed cost per kilogram of live weight gain by approximately 8–12%, demonstrating concurrent productivity and economic benefits. Similar results were reported by Roy et al. [14], who found that hydroponic maize increased feed intake by 6–9%, final body weight by 8–10%, and improved feed conversion ratio by 10–14%, reinforcing the evidence for enhanced growth efficiency.

Sulistijo et al. [13] introduced a novel approach by enriching hydroponic maize forage with fermented compost tea. Partial replacement of grass silage with this enriched hydroponic maize improved nutrient digestibility by 6–11% and increased total VFA production by 10–15%, without affecting DMI or blood metabolites including glucose, total protein, albumin, urea, AST, ALT, and total cholesterol at inclusion levels up to 75% DM. However, complete replacement reduced feed intake by 20–30%, indicating that even with supplementation, high substitution rates remain limiting.

In lactating goats, Marsico et al. [32] found that including hydroponically germinated oats at 1.5–3 kg fresh weight (10–20% DM) did not significantly affect milk production. Milk yield varied by less than ±2%, suggesting limited physiological impact under the tested conditions.

Research on sheep shows similarly variable responses depending on inclusion level and feeding context. Farghaly et al. [28] reported that feeding hydroponic barley sprouts as the sole forage reduced DMI by 15–25%, yet improved digestibility of OM and CP by 8–12% and increased total VFA by 10–14%. Rumen enzyme activity was also stimulated, indicating enhanced fermentative efficiency when hydroponic sprouts were fed alongside, but not replacing, a concentrate.

In contrast, Ansari et al. [29] showed that several hydroponic fodder varieties fed as sole diets resulted in low DMI (below maintenance), leading to negative nitrogen and mineral balances. These findings indicate that hydroponic fodder alone cannot meet the nutritional requirements of adult sheep, despite being economical to produce in some cases.

Guerrero-Cervantes et al. [31] evaluated hydroponic green wheat in gestating and lactating ewes and found that replacing dry-rolled corn and cottonseed meal had minimal impact on reproductive traits or DMI. Lamb birth weight and early growth were largely unaffected, and changes in blood metabolites were small (e.g., NEFA −8%, glucose −5%, BUN-10%), indicating that hydroponic wheat can partially replace conventional ingredients without impairing productivity (Table 4).

## 7. Growing Animals

Hydroponic forage has been evaluated across a wide range of production conditions in calves, beef cattle, goats, and sheep, generally showing benefits for rumen fermentation, nutrient utilization, and growth when included at moderate levels.

In calves, Benu et al. [12] reported that replacing grass silage with hydroponic maize forage reduced total dry matter intake by 10–18% but increased ruminal ammonia and total VFA concentrations by 12–20%, indicating improved fermentative activity despite the lower intake. Similarly, Rajkumar et al. [30] showed that including hydroponic maize at ~7% DM improved DMI, body weight gain, and feed efficiency, while reducing cost per kilogram of weight gain by approximately 8–10%, demonstrating both nutritional and economic advantages.

Evidence in beef cow-calf systems appears more limited. Crump et al. [11] found that supplementing sprouted barley at 12–13% DM had no significant effects on cow or calf performance, milk composition, or feed intake. Rumen fermentation patterns changed slightly over time, but these alterations did not translate into measurable improvements in growth, suggesting limited benefits at this inclusion level.

Studies in buffalo calves confirm the positive effect of hydroponic maize. Arif et al. [17] reported that replacing 40% of the basal diet with hydroponic maize increased DM and CP intake by 8–15%, enhanced digestibility by 6–9%, and improved feed conversion efficiency, confirming hydroponic maize as a viable option for improving growth performance in buffaloes.

Research on lambs has generated more consistent findings. Crump et al. [9] observed that substituting 10–30% of the diet with sprouted barley slightly reduced DMI (5–9%) but did not impair carcass characteristics. The 10% inclusion level yielded the lowest cost per unit of weight gain, suggesting a favorable economic threshold. Chetan et al. [19] demonstrated that very high inclusion of hydroponic maize (45–80% of the diet) progressively reduced DMI and ADG (from 77 g/d to ~21 g/d, −73%), although nutrient digestibility and blood parameters remained largely unaffected.

In Hu lambs, Ma et al. [15] identified an optimal inclusion range of 5–15% hydroponic barley sprouts, which improved milk composition (protein and fat) and enhanced lamb growth. Complementary results were reported by Min et al. [18], who found that including 15% hydroponic wheat sprouts increased DMI and weight gain by 12–18% and improved metabolic profiles, notably increases in HDL and IL-2, together with beneficial shifts in rumen microbiota (higher *Olsenella* and *Limosilactobacillus* abundance). These changes suggest improved immune status and rumen function.

Pasture-based systems also benefit from hydroponic supplementation. Mekonnen et al. [27] reported that adding hydroponic barley or oats to grazing diets increased DMI by 12–21%, prevented weight loss, and improved overall profitability, although practical constraints limit broad adoption. Similarly, Devendar et al. [26] found that replacing ~50% of concentrate protein with hydroponic barley sprouts increased DMI, ADG, and digestibility (DM, CP, NFE), while lowering production costs without altering carcass traits.

Sustainability-related outcomes have also been explored. Mpanza et al. [20] showed that including 25–50% hydroponic barley sprouts reduced methane emissions and improved rumen fermentation profiles, indicating potential environmental benefits. Rumen microbiome shifts toward more efficient fermenters further support the role of hydroponic sprouted grains in reducing the carbon footprint of lamb production.

Overall, the collective evidence indicates that hydroponic forage can enhance intake, digestibility, rumen fermentation, and growth in calves, goats, and sheep when included at moderate levels (5–20% DM). However, high inclusion rates (≥40–50% DM) frequently reduce DMI due to the high moisture content and potentially rapid rumen fill, limiting performance benefits despite improved fermentation. These patterns highlight the importance of defining species-specific and production-stage-specific inclusion thresholds to maximize the nutritional and economic value of hydroponic forage (Table 5).

## 8. Conclusions

This systematic review shows that hydroponic forage, particularly hydroponically sprouted barley, can be included in ruminant diets at moderate levels (10–20% DM) without negative effects on feed intake, milk yield, or growth performance. In dairy cows and buffaloes, low to moderate inclusion levels maintained DMI and milk yield, while in some cases increasing milk fat and antioxidant capacity.

In small ruminants, positive outcomes on ADG and feed efficiency were reported mainly when hydroponic forage replaced 20–40% of the basal diet, whereas high inclusion levels (≥50% DM) frequently reduced DMI and did not improve growth. Digestibility responses were inconsistent across studies, improving when hydroponic forage was used as a partial substitute but declining when inclusion exceeded 40–50% DM.

Evidence on rumen fermentation, blood metabolites, and immune responses indicates moderate physiological effects, but the magnitude varies across species and diets. Only one study evaluated methane emissions, showing a reduction, but current evidence is insufficient to draw conclusions regarding environmental impact.

Overall, hydroponic forage can be considered a partial substitute for conventional forages or concentrates, with optimal inclusion generally ranging between 10% and 30% of diet DM, depending on species and production stage.

However, the literature remains heterogeneous in terms of sprouting systems, growth duration, nutrient composition, and reporting standards. Future research should prioritize standardized methodologies, detailed nutrient and digestibility data, long-term production trials, and comprehensive evaluation of rumen microbiota and environmental outcomes, including methane emissions.

## Figures and Tables

**Table 1:** Descriptive summary of selected studies on the use of hydroponic forage in ruminants.

Reference	Year	Species	Breed	Hydroponic Crop	Country
[9]	2025	Lambs	Western White-Face	Barley	USA
[10]	2025	Buffalo	Mediterranean Italian Buffalo	Barley	Italy
[11]	2024	Cow/calves	Angus	Barley	USA
[12]	2024	Calves	Ongole x Brahman	Maize	Indonesia
[13]	2024	Goat	NA	Maize	Indonesia
[7]	2024	Goat	Bengal	Wheat	India
[14]	2023	Goat	Bengal	Maize	India
[15]	2023	Lamb	Hu	Barley	China
[16]	2023	Cow	NA	Barley	Italy
[17]	2023	Buffalo calves	Teddy x Local	Maize	Pakistan
[18]	2024	Lamb	Hu	Wheat	China
[19]	2022	Lamb	NA	Maize	India
[20]	2022	Lamb	Merino	Barley	South Africa
[21]	2022	Cow	Holstein	Barley	China
[22]	2024	Cow	Holstein	Barley	China
[23]	2024	Cow	Holstein	Barley and Wheat	China
[24]	2024	Buffalo	Mediterranean Italian Buffalo	Barley	Italy
[4]	2024	Buffalo	Mediterranean Italian Buffalo	Barley	Italy
[5]	2021	Cow	Holstein	Barley	Iran
[25]	2020	Cow	Holstein	Barley	South Korea
[26]	2020	Lamb	Deccani	Barley	India
[27]	2019	Lamb	Washera	Barley and Oat	Ethiopia
[28]	2019	Sheep	Barki	Barley	Egypt
[29]	2019	Sheep	Crossbred	Maize	India
[6]	2019	Cow	NA	Barley	Malta
[30]	2018	Calves	Crossbred	Maize	India
[31]	2016	Sheep	Katahdin	Wheat	Mexico
[32]	2009	Goat	Garganica	Oat	Italy

NA: not available.

**Table 2:** Descriptive statistics of the chemical composition of hydroponic barley, maize, and wheat forage.

Plant	DM	EE	CP	NDF	ADF	ADL	NSC	Starch	Ash
Barley	14.52	3.33	14.26	35.56	19.19	3.24	52.72	13.66	3.94
std	2.88	1.05	3.16	12.60	8.85	2.18	8.59	8.40	1.23
min	10.2	0.51	9.9	12.73	5.76	1.1	43.9	7.14	2.17
max	21.24	5.82	22.5	67.4	37.15	6.68	61.6	26	6.49
Maize	14.20	4.40	12.09	49.33	21.88	3.28	62.18	NA	3.36
std	3.20	1.02	1.52	23.09	9.02	2.43	8.41	NA	1.00
min	12.4	3.47	12.24	33	15.5	0.98	57.53	NA	2.73
max	17.26	5.82	13.2	65.66	28.26	5.82	71.89	NA	4.85
Wheat	16.25	2.63	16.2	38.35	16.735	1.48	NA	33.4	3.66
std	1.77	NA	1.84	26.09	12.82	0.39	NA	NA	NA
min	15.00	2.63	14.90	19.90	7.67	1.20	NA	33.40	3.66
max	17.50	2.63	17.50	56.80	25.80	1.75	NA	33.40	3.66

DM: dry matter; CP: crude protein; EE: ether extract; NDF: neutral detergent fiber; ADF: acid detergent fiber; ADL: acid detergent lignin; NSC: non-structural carbohydrates; std: standard deviation; min: minimum; max: maximum. NA: not available.

**Table 3:** Effects of hydroponic barley sprouts in dairy animals.

Study	Species	Parity	Inclusion (% DM)	DMI	Milk Yield	Milk Composition
[5]	Holstein cow	Multiparous	25–50%	↓ at high inclusion	↔	↔
[25]	Holstein heifer	Nulliparous	10–30%	↔	-	-
[22]	Holstein cow	Multiparous	10–20%	↓ ~0.5 kg/d	↔	↔
[23]	Holstein cow	Multiparous	10–30%	↓ 3–7%	(↑ at 10%)	↑ fat (+0.2–0.3%)
[16]	Holstein cow	Multiparous	15%	↔	↔	↑ fat (+0.2%)
[21]	Holstein cow	Multiparous	25–50%	↓ at 50%	↓ at 50%	↓ fat and protein
[6]	NA	10–20%	↔	↔	↔ milk	-
[4]	Buffalo	Multiparous	15%	↔	↔	↑ fat and protein
[24]	Buffalo	Multiparous	10–20%	↔	↔	↑ fat (+0.3–0.5%)

↑: increase; ↓: decrease; ↔: no effect. DM: dry matter; DMI: dry matter intake. NA: not available.

**Table 4:** (**A**) Effects of hydroponic forage in sheep. (**B**) Effects of hydroponic forage in goat.

Study	Breed	Inclusion (% DM)	DMI	Digestibility	Performance
(**A**)					
[31]	Katahdin	10–30%	↔	↔	↑ reproductive
[28]	Barki	25–50%	↔ or ↓	↑ OM, ↑ VFA	↑ fermentation
[26]	Deccani	10–30%	↑	↑ OM, ↑ CF	↑ ADG (+10–16%)
[27]	Washera	20–40%	↑	↔	↑ BW gain
[18]	Hu sheep	5–20%	↑ at 15%	↔ or ↑	↑ ADG (12–18%)
(**B**)					
[32]	Garganica	10–20%	↔	↔	↔ milk yield
[7]	Black Bengal	5–15%	↑	↑	↑ ADG, ↑ fertility
[13]	Kacang	30–75%	↓ at ≥60%	↑ OM, ↑ CF	↑ VFA, ↓ growth at 75%

↑: increase; ↓: decrease; ↔: no effect. OM: organic matter; VFA: volatile fatty acids; CF: crude fiber; BW: body weight; ADG; average daily gain.

**Table 5:** Studies on the use of hydroponic fodder in the feeding of growing animals.

Study	Species	Inclusion Level (% DM)	Performance
[12]	Calves	35–70%	DMI ↓ 10–18%; NH_3_-N ↑ 12–20%; total VFA ↑ 12–20%
[30]	Calves	~7%	↑ DMI, ↑ BW gain, ↑ FCR; ↓ cost/kg gain 8–10%
[11]	Cow–calf pairs	12–13%	↔ BW, DMI, milk
[17]	Buffalo calves	40%	↑ DMI +8–15%; ↑ CP intake; ↑ digestibility 6–9%; ↑ feed efficiency
[9]	Lambs	10–30%	DMI ↓ 5–9%
[19]	Lambs	45–80%	DMI and ADG ↓ 73%
[15]	Hu lambs	5–15%	↑ ewe milk protein and fat; ↑ lamb growth
[18]	Hu lambs	5–20%	DMI ↑ 12–18%; BW ↑ 12–18%; ↑ HDL, ↑ IL-2;
[27]	Sheep	10–20% (supplement)	DMI ↑ 12–21%
[24]	Lambs	~50% concentrate protein	↑ DMI, ↑ ADG, ↑ digestibility;
[20]	Lambs	25–50%	↓ methane

↑: Increase; ↓: Decrease; ↔ no effect. DMI: dry matter intake; ADG: average daily intake; BW: body weight; NH_3_-N: ammonia; VFA: volatile fatty acids; CP: crude protein; FCR: feed conversion ratio. HDL: High-Density Lipoprotein.

## Data Availability

No new data were created or analyzed during this study. Data sharing is not applicable.

## Abbreviations

The following abbreviations are used in this manuscript:

ADF	Acid Detergent Fiber
ADG	Average Daily Gain
ADL	Acid Detergent Lignin
BW	Body Weight
CF	crude fiber
CP	Crude Protein
DM	Dry Matter
DMI	Dry Matter Intake
EE	Ether Extract
FCR	Feed Conversion Ratio
HGF	Hydroponic Green Forage
HF	Hydroponic Forage
ME	Metabolizable Energy
NDF	Neutral Detergent Fiber
NH_3_-N	Ammonia Nitrogen
OM	Organic Matter
PUFA	Polyunsaturated Fatty Acids
RR	Replacement Rate
SFA	Saturated Fatty Acids
TMR	Total Mixed Ration
VFA	Volatile Fatty Acids
